# Reliability, Convergent Validity and Time Invariance of Default Mode Network Deviations in Early Adult Major Depressive Disorder

**DOI:** 10.3389/fpsyt.2018.00244

**Published:** 2018-06-08

**Authors:** Katie L. Bessette, Lisanne M. Jenkins, Kristy A. Skerrett, Jennifer R. Gowins, Sophie R. DelDonno, Jon-Kar Zubieta, Melvin G. McInnis, Rachel H. Jacobs, Olusola Ajilore, Scott A. Langenecker

**Affiliations:** ^1^Department of Psychiatry, University of Illinois at Chicago, Chicago, IL, United States; ^2^Department of Psychiatry and Behavioral Sciences, Northwestern University, Chicago, IL, United States; ^3^Department of Psychiatry, University of Utah, Salt Lake City, UT, United States; ^4^Department of Psychiatry, University of Michigan Medical Center, Ann Arbor, MI, United States

**Keywords:** depression, default mode network, cognitive control network, resting-state fMRI, time-invariance, reliability, rumination

## Abstract

There is substantial variability across studies of default mode network (DMN) connectivity in major depressive disorder, and reliability and time-invariance are not reported. This study evaluates whether DMN dysconnectivity in remitted depression (rMDD) is reliable over time and symptom-independent, and explores convergent relationships with cognitive features of depression. A longitudinal study was conducted with 82 young adults free of psychotropic medications (47 rMDD, 35 healthy controls) who completed clinical structured interviews, neuropsychological assessments, and 2 resting-state fMRI scans across 2 study sites. Functional connectivity analyses from bilateral posterior cingulate and anterior hippocampal formation seeds in DMN were conducted at both time points within a repeated-measures analysis of variance to compare groups and evaluate reliability of group-level connectivity findings. Eleven hyper- (from posterior cingulate) and 6 hypo- (from hippocampal formation) connectivity clusters in rMDD were obtained with moderate to adequate reliability in all but one cluster (ICC's range = 0.50 to 0.76 for 16 of 17). The significant clusters were reduced with a principle component analysis (5 components obtained) to explore these connectivity components, and were then correlated with cognitive features (rumination, cognitive control, learning and memory, and explicit emotion identification). At the exploratory level, for convergent validity, components consisting of posterior cingulate with cognitive control network hyperconnectivity in rMDD were related to cognitive control (inverse) and rumination (positive). Components consisting of anterior hippocampal formation with social emotional network and DMN hypoconnectivity were related to memory (inverse) and happy emotion identification (positive). Thus, time-invariant DMN connectivity differences exist early in the lifespan course of depression and are reliable. The nuanced results suggest a ventral within-network hypoconnectivity associated with poor memory and a dorsal cross-network hyperconnectivity linked to poorer cognitive control and elevated rumination. Study of early course remitted depression with attention to reliability and symptom independence could lead to more readily translatable clinical assessment tools for biomarkers.

## Introduction

Major Depressive Disorder (MDD) is a heterogeneous condition, which appears to have contributed to slow progress toward identifying endophenotypes for this disorder. Endophenotypes have characteristics of (1) being associated with the illness, (2) being present prior to, during and after an episode, thus relative state independence, (3) heritability, (4) co-segregation with illness within families, (5) a greater representation in unaffected family members relative to the general population, and (6) must demonstrate good psychometric properties, including reliability (1, 2). The importance of state independence, including sensitivity in remitted states of the illness, is often overlooked in studies of endophenotypes. Because state effects of illness could obscure effective and reliable measurement, it is an important criterion to address in the search for endophenotypes of MDD (3).

One candidate endophenotype for depression has been increased resting-state connectivity within the default mode network (DMN) or between DMN and other nodes [e.g., (4)]. Resting-state functional MRI (rs-fMRI) is a useful technique for testing network efficiency and function. By examining temporal correlations of spontaneous low-frequency fluctuations in blood oxygen-level dependent signals, individual functional connectome maps and seed-to-node or seed-to-network patterns can be observed. rs-fMRI has many advantages—it does not rely on explicit task-based performance, is technically easy to collect, has higher signal-to-noise ratios, reduces participant burden, and can be analyzed remotely (5–7). This technique might be uniquely poised to identify clinically meaningful and stable deviations in MDD.

Within MDD, aberrant resting-state connectivity has been observed in the DMN, a network characterized as a set of regions coordinated in activity during mind-wandering, passive background thoughts, or rest (8, 9). Aberrant DMN connectivity has in turn been linked with clinical correlates of the illness [e.g., (10, 11)]. Furthermore, the DMN is likely involved in self-referential activities such as episodic memory, future planning, ruminative thought, and stimulus salience evaluation activities, many processes implicated in depression (12, 13). For example, spontaneous DMN hyperconnectivity in MDD is thought to reflect an over-attendance to internal self-relevant stimuli (10, 14). This network provides an opportune window into understanding how brain-based measures relate to depression etiology.

A number of challenges remain in defining the limits and specificity of disrupted DMN connectivity in MDD (1). Apart from the criteria of disease association, little other research has evaluated and validated DMN hyperconnectivity as an endophenotype, such as reliability, state independence, co-segregation or heritability. Some treatment studies predicting remission and measuring changes after treatment have pointed to changes in DMN connectivity associated with treatment response [see (14, 15) for review, and (16)]. Unfortunately, to our knowledge, few studies of remitted MDD exist, and no studies have examined stable, altered DMN connectivity across mood states, which would be a conservative test of state independence.

There are a few studies of resting-state DMN functional connectivity in remission from depression compared to healthy controls. One recent study by our group (with a smaller subset of the current sample) demonstrated increased left posterior cingulate cortex (PCC) to right middle frontal gyrus (MFG) connectivity present in both active MDD and remitted MDD (rMDD), suggesting state independence (17). Another study reported increased PCC to dorsolateral prefrontal cortex connectivity in active depression (18). A large family study demonstrated similar dysconnectivity in family members with shared genetic risk but no expression of illness, adding an important necessary feature for establishing an endophenotype (19). Older adults in remission showed over time a greater decline of connectivity between left hippocampal and posterior cingulate cortex, and greater increased connectivity between right hippocampus and prefrontal regions (20), which were both associated with cognitive functioning decline over time. One other small sample failed to find significant differences in DMN between rMDD and healthy controls after neutral mood induction (21). A recent meta-analysis highlighted that DMN hyperconnectivity patterns are fairly universal in active MDD (14), although at least one study reported hypoconnectivity in a larger sample (22) and another reported no differences within DMN (17).

While these numerous studies make important contributions to the validation of DMN hyperconnectivity as a marker for MDD risk, no study has yet evaluated the test-retest reliability criterion for these network disruptions to establish that a particular measurement does not vary by day, mood state, or depressive symptom manifestations. Test-retest reliability is considered acceptable if relative group rank on a variable remains similar over time. However, test-retest reliability does not reflect whether there is exact equivalence in a measurement over time (e.g., stability of exact numerical measurement across multiple time points). It is as yet unclear whether these rs-fMRI disruptions would meet some of the strict criteria for an endophenotype, including test-retest reliability over time.

Apart from the sparse links to heritable risk patterns, there is also a paucity of research determining whether rs-fMRI shows illness-related state effects (e.g., current mood), burden effects (e.g., depressive scars), and/or trait, risk, or disease aspects [e.g., state independence; but see (22)]. These limited associations currently studied with rs-fMRI have stalled understanding and application in translational research. Observation of rs-fMRI connectivity patterns independent of mood symptoms, such as measured during remission from depression, has an additional advantage: consistent differences between rMDD and never-depressed individuals might also be sensitive to reliable markers of illness. To investigate reliable markers of illness, confounding effects of repetitive illness scarring (e.g., additional episodes and morbidity) and developmental variability in brain maturation are best if diminished in influence. Examining those in the early course of illness reduces these confounds as does examining young adults nearing the end of their brain-based developmental trajectory (23). It is important to note that state effects (e.g., within an episode) could obscure or even invert patterns of connectivity that are most prescient for clinical utility: prediction of risk, course of illness, and morbidity (17, 24).

Disrupted connectivity patterns may underlie, compensate for, or reflect the outcome of other illness features such as abnormal behavior, performance, or personality characteristics. Brain-based markers may also exist free of the limitations of awareness and the capacity to report internal experiences. Evaluating links between network functioning and cognitive features could offer insight for reduced-risk treatments and disease marker modifications. Several DMN-relevant cognitive markers, including rumination, episodic memory, cognitive control, and emotional processing, are consistently different between depressed and healthy samples [e.g., (25)]. For instance, maladaptive passive rumination has been linked to greater dominance of the DMN over other networks (10), and when induced during fMRI, activates PCC, medial prefrontal cortex, and parahippocampus to a greater extent in currently depressed adults compared to controls (26). Prior studies from the longitudinal sample reported in the current study have documented deficits in young adults with rMDD, including increased rumination (27), performance deficits in delayed cued recall and recognition (28), decrements in cognitive control (3) and more accurate identification of sad and happy facial emotions (29).

**Figure 1:**
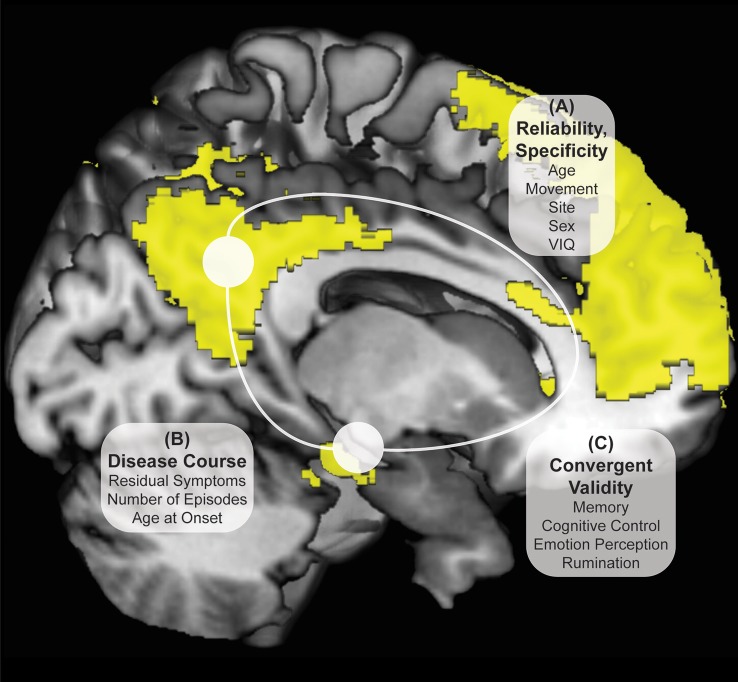
Investigation Strategy for DMN Reliability and Time Invariance in MDD. Yellow DMN viewed from medial sagittal slice. White circles denote PCC and HPF seeds. Analyses examine DMN connectivity differing between rMDD and HC for **(A)** reliability and specificity by examining ICCs after controlling for numerous potential confounders; **(B)** disease course by examining the remitted phase and clinical features; and **(C)** exploratory convergent validity through correlation with cognitive features of the illness.

The current study seeks to demonstrate that DMN connectivity abnormalities in individuals with MDD are reliable and illness course-independent by examining the stability of any hyper- and hypo- connectivity from the DMN in a larger sample, across 2 sites, in the remitted phase of depression, within a tightly controlled young adult sample (see Figure 1). In addition, the study examines whether group differences in connectivity are associated with cognitive features demonstrated in the remitted phase in prior work. We expect to find time-invariant DMN hyperconnectivity in rMDD with moderate reliability, such as between bilateral PCC and dorsolateral prefrontal cortex. In addition, we expect strong reliability across diagnostic groups in core connectivity within the DMN, such as PCC and anterior hippocampal formation (HPF) connectivity with orbitofrontal regions of prefrontal cortex. Finally, we expect DMN connectivity from all seeds with other DMN regions to be related to ruminative style and memory performance, and possibly inversely with facial emotion identification and cognitive control performance.

## Materials and methods

### Participants

This study was approved by the University of Michigan (UM) and the University of Illinois at Chicago (UIC) Institutional Review Boards. Participant diagnoses (both HC and rMDD) were determined by masters or doctoral-level clinicians using the Diagnostic Interview for Genetic Studies with participants, and confirmed with a parent or sibling using a modified Family Interview for Genetics Studies (30). All participants were excluded for current active depressive episode, current antidepressant and related psychotropic medication(s), and any suicidal attempts, or substance abuse or dependence, including tobacco and alcohol, within the last 6 months. Healthy controls (HCs) were excluded from participation if they met current or past criteria for any Axis I or II DSM-IV-TR psychiatric disorder or had a first-degree relative with a history of psychiatric illness. Participants with rMDD were not excluded for comorbid diagnosis of anxiety disorder, considering substantial overlap of symptoms and frequent comorbidity. The final sample included 47 individuals with rMDD (16 UM) and 35 HCs (13 UM) between 18 and 23 years of age at time of intake (UM: 19 females, UIC: 36 females) with 2 separate rs-fMRI scans (see Table 1).

**Table 1:** Demographic and Clinical Characteristics of Sample.

	**HC (*****n*** = **35)**		**rMD (*****n*** = **47)**		**Significance test**
**Characteristic**	***M***	***SD***	***M***	***SD***	
Gender (M/F)	14/21		13/34		$\chi_{\left(82\right)}^{2}$ = 1.38
Handedness (L/R)	2/33		4/43		$\chi_{\left(82\right)}^{2}$ = 0.23
Age at First Scan	21.45	1.67	22.16	1.53	*t*_(80)_ = 1.97
Years Education	14.54	1.36	14.66	1.29	*t*_(80)_ = 0.40
Verbal IQ Estimate	105.86	9.27	106.67	10.48	*t*_(80)_ = 0.25
HAM-D^a^^,^^c^	0.43	1.01	1.64	2.11	*t*_(72.86)_ = 3.54^*^
RRS Total^b^^,^^c^ (*N* = 68)	29.00	8.65	46.68	16.11	*t*_(62.45)_ = 5.85^*^
CC Factor^d^ (*N* = 70)	0.26	0.84	−0.01	0.98	*t*_(68)_ = 1.15
SLLT Hit d'^d^ (*N* = 81)	3.60	1.58	3.73	1.64	*t*_(79)_ = 0.41
FEPT Happy Accuracy^d^ (*N* = 71)	93.15%	7.21%	94.73%	8.00%	*t*_(69)_ = 0.92
FEPT Sad Accuracy ^d^	76.25%	15.76%	76.09%	12.78%	*t*_(69)_ = 0.001
Age of Onset			16.61	3.45	
Years in Remission			2.72	1.72	
Number Previous Episodes			1.76	1.12	

*Verbal IQ estimate was obtained with vocabulary subtest of the Shipley Institute of Living Scale. CC, Cognitive Control; FEPT, Facial Emotion Perception Test; HAM-D, Hamilton Depression Rating Scale; HC, Healthy Control; rMDD, Remitted Major Depressive Disorder; RRS, Ruminative Response Scale; SLLT, Semantic List Learning Task*.

a *g_Hedges_, 0.69*.

b *g_Hedges_, 1.30*.

c *Levene's test significant, thus degrees of freedom are adjusted*.

d *Prior studies with this sample demonstrated significant between-group differences. As this sample was smaller, accounting for attrition between first and second scans, some results are not significant herein*.

* *p < 0.05, all two-tailed*.

### Procedures

**Figure 2:**
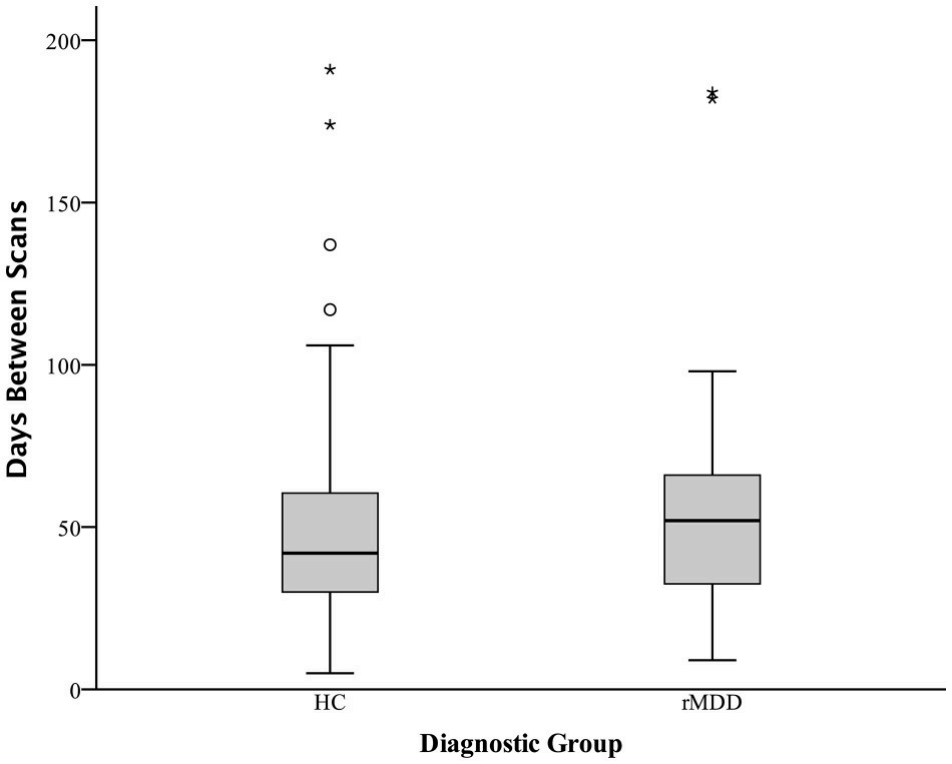
Days Between fMRI Scans Boxplot Separated by Diagnostic Group. Boxplots of number of days between two fMRI scan sessions for healthy control and remitted major depressive disorder participants. Dark colored line represents the median, open circles are outliers and asterisks represent significant outliers. There were a total of 4 outliers for healthy controls and 2 outliers for remitted MDD individuals.

Participants were screened over the phone. After explanation of study details, written informed consent was obtained. Masters or doctoral-level clinicians conducted Diagnostic Interviews for Genetic Studies (30) to determine prior diagnosis, current remission from MDD, and residual depressive symptoms [HAM-D; (31)]. Participants then completed the Rumination Responses Scale [RRS; (32)] and Parametric Go/No-Go Test (33), and during the first MRI scan, completed the Facial Emotion Perception Test [FEPT; (33)] and Semantic List Learning Test [SLLT; (34)]. One resting-state scan was taken at this first session and one at the second session, scheduled at a later time convenient for participants (in days: M = 54.57, *SD* = 37.79; typically between 4 and 12 weeks, see Figure 2). Analyses include all subjects without motion issues with available data; Little's MCAR test suggests that all missing variables from self-reports and tasks are missing at random ($\chi_{\left(74\right)}^{2}$ = 81.46, *p* = 0.26).

### fMRI acquisition

An eyes-open resting-state scan was acquired over 8 min with 3.0T GE scanners (Milwaukee, WI) using T2-weighted sequences with TRs of 2,000 ms length and 240 TRs in total. At UM, an eyes-open resting state scan was acquired over 8 min on a 3.0T GE Signa scanner (Milwaukee, WI) using T2-weighted single shot reverse spiral sequence with the following parameters: 90° flip, field-of-view = 20, matrix size = 64 × 64, slice thickness = 4 mm, 30 ms echo time, 29 slices. At UIC, eyes-open, resting scans were collected over 8 min on a 3.0T GE Discovery scanner (Milwaukee, WI) using parallel imaging with ASSET and T2 gradient-echo axial EPI with the following parameters: 90° flip, field-of-view = 22, matrix size = 64 × 64, slice thickness = 3 mm, 22.2 ms echo time, 44 slices. At both sites, high-resolution anatomic T1 scans were obtained for spatial normalization and calculation of gray matter volume (GMV) estimates. At UM, these T1 MPRAGE fast gradient echo images were recorded with the following parameters: 90° flip angle, field-of-view = 20, matrix size = 256 × 256, slice thickness = 4 mm, and 29 slices. At UIC, T1 SPGR images were recorded with the following parameters: 13° flip, field-of-view = 22, matrix size = 256 × 256, slice thickness = 1 mm, acquiring 186 slices. Motion was minimized with foam pads, a visual tracking line (UIC only) and/or fixation cross (UIC and UM) on the display, and by conveying the importance of staying still to participants, with TRs of 2,000 ms length and 240 TRs in total for the scan session at each site. Eight-min scans were conducted to maximize intersession reliability while ensuring low participant burden and head motion (35). Scan characteristics are presented in Table 2.

**Table 2:** Movement deviation adjustments and days between scans in the current sample.

	**HC (*****n*** = **35)**		**rMDD (*****n*** = **47)**	
	**M/N**	**SD/%**	**M/N**	**SD/%**
**SUBJECT SCAN CHARACTERISTICS**				
Site (UM)	13	37%	16	34%
Days Between Scans^a^	54.46	42.12	54.66	34.68
**Scan 1 (mm)**
x translation	0.19	0.16	0.16	0.19
y translation	0.05	0.05	0.05	0.04
z translation	0.05	0.05	0.06	0.05
**Scan 2 (mm)**
x translation	0.15	0.14	0.17	0.14
y translation	0.04	0.04	0.05	0.05
z translation	0.05	0.04	0.05	0.05
**MOVEMENT DEVIATIONS RELIABILITY OVER TIME (ICC)^b^**				
x translation	0.30^c^		0.45	
y translation	0.82		0.48	
z translation	0.47		0.60	

*UM, University of Michigan. Movement deviations are the standard deviations of the realignment adjustments over the 8-min resting state scan*.

a *Levene's test significant at p < 0.05*.

b *Reliability of movement ICC for all subjects was 0.38, 0.66, and 0.56^c^ for pitch, roll, and yaw, respectively*.

c *Not significantly different from zero at p < 0.05*.

### Subject movement exclusions

Several steps were taken to ensure subject movement did not have an undue influence on the current analyses. Motion of 1.5 mm or more in any direction over 3 consecutive TRs was used as a gross criterion for participant exclusion from analyses (36, 37); any TR to TR movement exceeding 0.5 mm was also basis for exclusion. This movement did not differ between groups (37). Finally, greater than 2 mm movement over an entire 8-min scan, or evidence of outlier status as a movement deviation value across the entire time series in relation to the rest of the sample was also used as a criterion for participant exclusion from current analyses. Equal numbers of HC and rMDD are identified and removed using these procedures, including in this sample [$\chi_{\left(69\right)}^{2}$ = 1.52, *p* = 0.22; see Figure 2]. Notably, motion scrubbing was not used.

### Seed selection and functional network determination

Bilateral seeds were derived from previous literature examining DMN resting-state connectivity of the PCC (38, 39) and HPF (34, 40), using the following coordinates: ±5, −50, 36 (PCC), ±30, −12, −18 (HPF). Data-driven network definitions typically require larger sample sizes for stability, thus we used empirically determined and validated seeds to test our hypotheses. Regions of Interest (ROIs, 2.9 mm radius, 19 voxels) were defined in Montreal Neurological Institute (MNI) space. Seeds were overlaid on the average warped structural anatomy of the current sample to determine accuracy in seed location.

Significant clusters from analyses were overlaid on functional masks of 5 networks, specifically DMN, Cognitive Control Network (CCN), Salience Emotion Network (SEN), Somatomotor Network, and Visual Network to identify likely network belonging (41). The CCN was created by addition of dorsal attention and frontoparietal networks, and the SEN was created by addition of ventral attention and limbic networks from an initial 7-network model to create a well-known 3 network model (4).

### fMRI preprocessing

Several steps were taken to reduce potential sources of noise and artifact as well as alignment with MNI template for uniform reporting. Slice timing was completed with SPM8 (http://www.fil.ion.ucl.ac.uk/spm/doc/, R4667) and motion detection algorithms were applied using FSL (http://fsl.fmrib.ox.ac.uk/fsl/fslwiki/, version 5.1). Coregistration of structural images to functional images was followed with spatial normalization of the coregistered T1-spgr to the MNI template. The resulting normalization matrix then was applied to the slice-time-corrected, physiologically corrected time series data. These normalized T2 time series data were spatially smoothed with a 5 mm Gaussian kernel resulting in T2 images with isotropic voxels, 2 mm on each side. Gray matter volume was estimated following segmentation with DARTEL (VBM within SPM8) and application of a 8 mm Gaussian kernel and conversion to 2 mm isotropic voxels. Gray matter volume was not significantly different between rMDD relative to HC [*F*_(1, 66)_ = 2.78, *p* = 0.08] and was lower in UIC relative to UM [*F*_(1, 66)_ = 5.62, *p* = 0.02]. Males had larger volumes than females [*F*_(1, 66)_ = 5.36, *p* = 0.02].

Time series data were detrended and mean-centered. Physiologic correction was performed by regressing out white matter and cerebral spinal fluid signals (42), as were motion parameters (36). Global signal regression and motion scrubbing were not conducted due to collinearity violations with gray matter signal, problematic mis-estimates of anticorrelations (43), and distortion of distance-micromovement relationships (36, 44, 45). Time-series were band-pass filtered over 0.01–0.10 Hz. Movement was also addressed using regression of white matter and cerebrospinal fluid signals (36, 37). Correlation coefficients were calculated between mean time course for seed regions and all other voxels of the brain, resulting in a 3-dimensional correlation coefficient image. These r images were Fisher transformed to *z-*scores.

Resulting *z* images were used in a 2 (Group) by 2 (Time) repeated-measures analysis of covariance (ANCOVA) implemented in SPM8, controlling for sex, movement translation (in x, y, and z translations) and site. Up-to-date AFNI 3dClustSim was used to evaluate correction using Monte Carlo simulations (1000 iterations). Bayesian whole-brain correction of *p* = 0.01 is achieved with a joint threshold of height and extent (*k* > 57, *p* < 0.005, 440 mm^3^) for each seed-based *F*-test analysis for a family-wise error rate for 4 analyses at *p* < 0.04. Additional scan characteristics are presented in Table 2.

### Reliability, convergent validity and disease course analyses

A whole brain 2 (Group) × 2 (Time) ANCOVA was conducted in SPM8 for each seed. Averaged time series correlations from each seed at each scan session extracted from each significant cluster identified in the *F*-test were used to test reliability (primary hypothesis), convergent validity (added for *post-hoc* analyses to understand clinical relevance), and disease course (potential nuisance factors in identifying endophenotypes) associations. For reliability, due to non-random assignment to MRI scanners, and no scans conducted at both sites for any participants, one-way random analyses to quantify intraclass correlation coefficients (ICCs) were run for each significant cluster across all participants. These ICCs were also quantified for each diagnostic group to assess diagnostic differences in reliability and are reported in Table 3. Because of the numerous regions found in these repeated-measures ANCOVAs, several steps were taken to reduce the number of additional tests, and thus Type I error rates, needed to examine convergent validity and disease course. First, these significant clusters were averaged across scan time points. Second, principle component analysis (PCA) was conducted on scan-averaged connectivity values for each cluster, and then rotated with an oblique promax rotation.

**Table 3:** Connectivity differences between rMDD and HC with default mode network seeds.

**Seed with cluster^a^**	**Network**	**Peak MNI coordinates**	**Cluster size (*k*)**	**Intensity (*F*)**	**g**_**Hedges**_		**ICC^b^**		
					**T1**	**T2**	**All**	**HC**	**rMDD**
**rMDD** > **HC**									
**L-PCC Seed**
R-SFG	DMN/SEN	8, 16, 62	168	21.03	0.69	0.82	0.65	0.62	0.56
L-MFG	CCN	−38, 44, 16	113	13.22	0.60	0.71	0.64	0.69	0.51
R-MFG	DMN/CCN	32, 32, 42	157	17.69	0.82	0.79	0.73	0.62	0.71
B-Prec	CCN	−2, −70, 46	334	16.30	0.78	0.94	0.66	0.66	0.50
**R-PCC Seed**									
R-SFG	CCN	32, 10, 56	67	14.45	0.53	0.80	0.73	0.63	0.74
L-MFG	CCN	−38, 46, 16	173	13.38	0.58	0.66	0.73	0.83	0.53
R-MFG	CCN	42, 36, 22	556	21.37	0.85	0.90	0.73	0.80	0.49
L-IPL	CCN	−34, −58, 34	137	16.38	0.60	0.81	0.50	0.19	0.51
R-IPL	CCN	44, −52, 42	354	19.09	0.54	1.01	0.76	0.69	0.73
B-Prec	CCN	−4, −70, 44	332	19.76	0.77	0.81	0.70	0.69	0.60
**rMDD < HC**									
**R-PCC Seed**									
B-Prec	DMN	2, −52, 32	120	16.34	−0.68	−0.45	0.70	0.76	0.55
**L-HPF Seed**									
R-STG	SMN/DMN	66, −34, 8	176	15.27	−0.77	−0.41	0.67	0.50	0.72
**R-HPF Seed**									
R-mOFG	DMN	10, 60, −6	67	12.97	−0.75	−0.29	0.73	0.69	0.73
L-mOFG	DMN/SEN	−6, 56, −6	74	12.34	−0.53	−0.38	0.75	0.78	0.68
L-STG	SEN	−28, 14, −32	100	16.24	−0.80	−0.76	0.59	0.63	0.37
R-STG	SMN/DMN	68, −16, −2	219	22.25	−0.91	−0.42	0.72	0.68	0.70
R-Para	SMN	8, −42, 66	241	16.39	−0.97	−0.69	0.37	0.13	0.20

*B, Bilateral; CCN, Cognitive Control Network; DMN, Default Mode Network; g_Hedges_, unbiased Hedges g; HPF, Hippocampal Formation; ICC, Intraclass Correlation; IPL, Inferior Parietal Lobule; MFG, Middle Frontal Gyrus; mOFG, Medial Orbitofrontal Gyrus; Para, Paracentral Lobule; PCC, Posterior Cingulate Cortex; Prec, Precuneus; SEN, Salience Emotion Network; SFG, Superior Frontal Gyrus; SMN, Somatomotor Network; STG, Superior Temporal Gyrus*.

a *All reported clusters significant at p < 0.005, k > 57. Seed ROIs: radius, 2.9 mm; location, PCC ±5, −50, 36 and HPF ±30, −12, −18*.

b *One-way random, average measures, ICC (1, 2)*.

Clusters for the following analyses were not chosen *a-priori*, and PCA factors were data-driven, thus two-tailed correlations with uncorrected significance were chosen to explore relationships with clinical and cognitive features. These correlations were conducted to assist in interpretation of any between-group differences and did not serve as primary hypotheses. As these are exploratory descriptive analyses, and because seed-network connectivity differences are likely derived from multifactorial processes, we did not expect robust associations and thus did not adjust for multiple comparisons. We investigated disease severity, disease course, and demographics to identify any nuisance variables. Disease course variables included residual depressive symptoms [HAM-D; (31)], number of depressive episodes, age at onset of illness, and demographic variables included verbal intelligence (46) and age at first scan. Cognitive features included ruminative tendencies (27), a cognitive control factor (obtained by principal components analysis with oblique rotation of percent correct inhibitory trials from the Parametric Go/No-Go task) (3), happy and sad accuracy from the FEPT (29), and memory sensitivity (e.g., hits) on the SLLT (28, 34, 47). Follow-up partial correlation analyses controlling for diagnosis were conducted on significant correlations to determine if any relationships between clinical or cognitive features and connectivity were independent of diagnosis.

### Exploratory state independence comparisons with active and familial risk for depression

**Figure 3:**
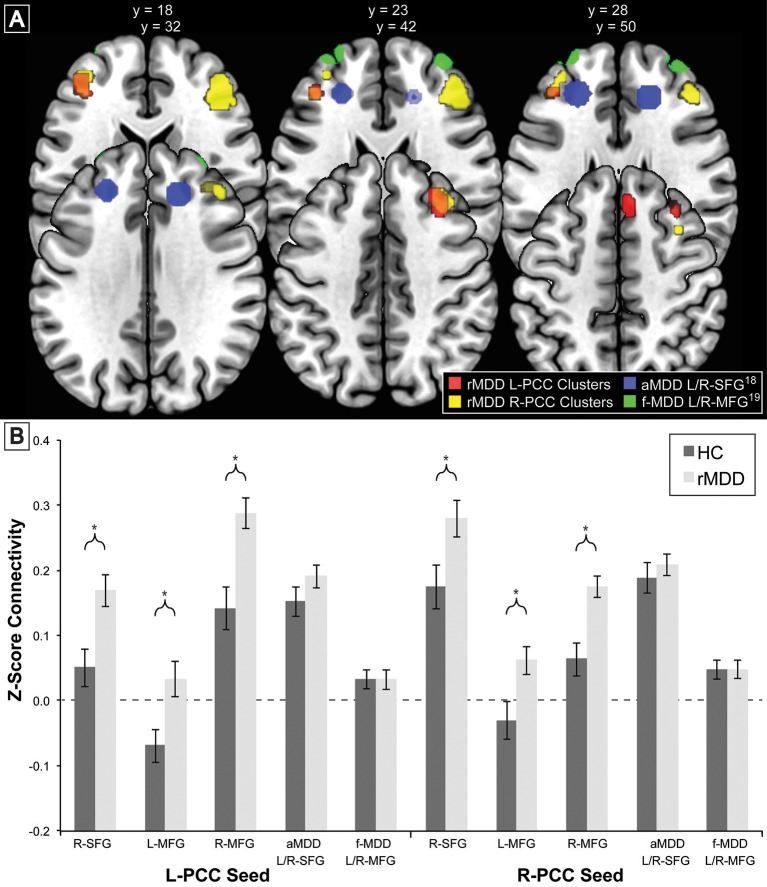
Comparison of Prefrontal Cortex Clusters with Active and Familial Risk for Depression. Comparison of 3 left PCC seed rMDD-different connectivity clusters (red), 3 right PCC seed rMDD-different connectivity clusters (yellow), bilateral SFG connectivity from precuneus seed (also showing connectivity with our PCC seed) found in active depression (18) (blue) and bilateral MFG connectivity from PCC found in familial-risk for depression (19) (green). **(A)** Axial view of each significant cluster and ROIs with 8 mm radius found in studies of active and familial-risk for depression. **(B)** *t*-test comparisons of mean connectivity in first scan from bilateral PCC to prefrontal cortex clusters found in the current study, combined left and right active depression SFG, and combined left and right familial-risk depression MFG. Bars represent ±1 standard error from the mean. ^*^Significantly different between groups (*p* < 0.005, *k* > 57). aMDD, active major depressive disorder; f-MDD, familial-risk for major depressive disorder.

To compare our findings of significant differences in cross-network correlations between PCC and prefrontal cortex in rMDD with previous similar findings for an active depression sample (18) and a high familial-risk for depression sample (19), bilateral spherical ROIs in the prefrontal cortex with an 8 mm radius were derived from these studies' reported peak coordinates of group differences in connectivity from precuneus: ±7, −60, 21 (18), and PCC: ±9, −85, 37 (17, 19). Notably, active depression demonstrated connectivity between precuneus and dorsolateral prefrontal cortex as well as between dorsolateral prefrontal cortex and areas including our ROI seeds within bilateral PCC. Mean time cluster values for each participant and scan from bilateral PCC were used to compare connectivity between the current rMDD and HC sample using a linear mixed modeling approach to control for inherently correlated connectivity within each participant. These ROIs as well as the diagnostically-different connectivity clusters found in the current sample (Table 1) are shown in Figure 3A. Appropriate number of variance components and covariance structure were identified by comparing Akaike's information criteria (AIC) across models to find the best model to explain variance, fit the data, and reduce complexity. A coherent model with the primary SPM analytic model was first constructed prior to reduction of model complexity (48). Included in the best-fit final model was the intercept representing male HCs from UM at the first scan and fixed factors of diagnosis, scan session (heterogenous first-order autoregressive covariance structure), diagnosis by scan session, sex, site, and a random subject-specific intercept, along with random covariates of x, y, and z translation in a diagonal covariance structure.

## Results

### Potential clinical and demographic confounds

To ensure diagnostic findings were not unduly influenced by demographic and technological factors, diagnostic group differences on technical variables were examined. These variables can be seen in Table 2. Scanning site was evaluated as an inadvertent source of group differences, although the greater likelihood is that multiple scanning sites will lead to weaker power to test hypotheses and thus greater Type II errors. Between-site differences could potentially bias results observed, despite current assumptions that rs-fMRI is relatively impervious to local scanner specifics. The percentage of individuals scanned at each site within each diagnostic group was not significantly different [$\chi_{\left(1,\;82\right)}^{2}$ = 0.08, *p* > 0.10]. Nonetheless, site was still included as a covariate of non-interest.

Standard deviations of subject movement were not significantly different between diagnostic groups [*x*: *t*_(80)_ = 0.10, *p* > 0.10; *y*: *t*_(80)_ = −0.46, *p* > 0.10; *z*: *t*_(80)_ = −0.72, *p* > 0.10], but were included as covariates of non-interest according to standard fMRI analytic procedures. The number of days between scans was not significantly different between groups and thus was not included as a covariate [*t*_(80)_ = −0.02, *p* > 0.10].

### Time-invariant diagnostic differences

**Figure 4:**
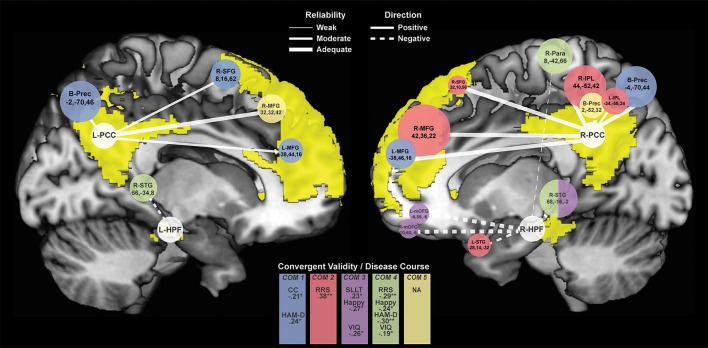
Connectivity Clusters Different Between rMDD and HC from Bilateral PCC and HPF. White circles denote bilateral seeds on medial sagittal view of each hemisphere with DMN highlighted in yellow. Branches to clusters are weighted by level of reliability, with solid branches representing hyperconnectivity in rMDD and dotted branches representing hypoconnectivity. Cluster size is scaled according to number of significant voxels. Colors indicate component-belonging and significant/trend-level convergent validity and disease course associations. COM, Component. ^†^*p* < 0.10, ^*^p < 0.05, ^**^p < 0.01.

Seventeen connectivity clusters significantly differed between HC and rMDD at the *F*-test level from the 4 seed ROIs, demonstrating stability. Figure 4 illustrates the connections of these 17 clusters and illustrates relationships of these components to cognitive features to examine clinical meaningfulness. Table 3 shows significant clusters with covariates controlling for site, sex, and movement translations.

Compared to HCs, rMDD individuals showed greater connectivity within DMN and between DMN and CCN. For example, connectivity was greater between left PCC seed and bilateral middle FG and posterior dorsal precuneus, as well as between right PCC seed and right superior frontal gyrus (SFG), bilateral middle FG, bilateral inferior parietal lobule, and bilateral precuneus (Figure 4). rMDD individuals also demonstrated greater connectivity within DMN, specifically between left PCC seed and right SFG.

In contrast, compared to HCs, rMDD demonstrated lower connectivity between DMN, SEN and the somatomotor network (SMN), specifically between right PCC seed and bilateral precuneus, between left HPF seed and right superior temporal gyrus, and between right HPF and bilateral superior temporal gyrus, bilateral medial orbitofrontal gyrus (mOFG) and right paracentral lobule.

ICCs (Table 3, Figure 4) showed 1 weak (*r* = 0.30–0.49), 6 moderate (*r* = 0.50–0.69), and 10 adequate (*r* = 0.70–0.79) reliable connectivity cluster differences across groups. No reliabilities met criteria for good (*r* = 0.80–0.89) or strong effects (*r* > 0.90).

### Data reduction by PCA

Five components were identified by PCA, explaining 71.11% of the variance for connectivity of the 17 clusters averaged across scans (extractions > 0.40). Component loadings, as identified in the pattern matrix, are reported in Table 4. The first component was primarily composed of positively-loading clusters in CCN with greater connectivity to bilateral PCC in rMDD—Bilateral PCC with CCN. The second component positively-loaded on right PCC seed with clusters in right frontal gyri and bilateral inferior parietal lobule greater in rMDD—Right DMN with CCN and SEN. The third component consisted of positively-loaded connectivity clusters greater in HC from right HPF seed with bilateral frontal gyri and right superior temporal gyrus—Right HPF with DMN. The fourth component consisted of positively-loaded bilateral HPF seed with right superior temporal gyrus and right HPF seed with ipsilateral paracentral lobule—HPF with SMN. Finally, component 5 consisted of positively-loaded left PCC seed with right MFG and right PCC seed with bilateral precuneus—PCC with DMN. Components 1 and 2 were significantly greater in rMDD than HC [*t*_(80)_ = −5.28, *p* < 0.001; *t*_(80)_ = −5.30, *p* < 0.001, respectively], components 3 and 4 were greater in HC [*t*_(80)_ = 2.10, *p* = 0.04; *t*_(80)_ = 5.95, *p* < 0.001, respectively], and the last component did not significantly differ by diagnosis [*t*_(80)_ = 0.37, *p* = 0.71; see Table 4].

**Table 4:** Loadings for connectivity components derived from PCA.

	**Connectivity PCA component**				
**Seed with cluster**	**1: Bilateral PCC with CCN**	**2: Right DMN with CCN/SEN**	**3: R-HPF with DMN**	**4: HPF with SMN**	**5: PCC with DMN**
**rMDD > HC**					
**L-PCC Seed**					
R-SFG	**0.46**	0.22	−0.05	−0.10	0.01
L-MFG	**0.89**	−0.14	0.05	0.03	−0.21
R-MFG	0.18	0.23	0.20	−0.39	**0.63**
B-Prec	**0.95**	−0.20	−0.18	0.12	0.31
**R-PCC Seed**					
R-SFG	−0.23	**0.92**	−0.10	−0.06	0.14
L-MFG	**0.83**	0.07	0.15	−0.03	−0.26
R-MFG	0.35	**0.53**	0.08	−0.14	−0.09
L-IPL	0.22	**0.68**	0.04	0.06	−0.06
R-IPL	0.01	**0.87**	−0.09	0.10	0.07
B-Prec	**0.73**	0.16	−0.21	0.09	0.16
**rMDD < HC**					
**R-PCC Seed**					
B-Prec	−0.16	0.06	0.01	0.30	**0.75**
**L-HPF Seed**					
R-STG	0.10	0.15	0.33	**0.77**	0.05
**R-HPF Seed**					
L-mOFG	−0.10	−0.03	**0.92**	−0.02	0.09
R-mOFG	−0.04	−0.18	**0.90**	−0.05	0.05
L-STG	0.09	−**0.42**	0.32	0.001	0.34
R-STG	0.01	0.16	**0.51**	**0.65**	0.0001
R-Para	0.03	−0.17	−0.25	**0.75**	0.06
**DIAGNOSTIC DIFFERENCES BY COMPONENT**					
HC *M* (SD)	−0.59 (1.02)	−0.59 (0.83)	0.26 (1.10)	0.64 (0.76)	0.05 (1.08)
rMDD *M* (SD)	0.44 (0.73)	0.44 (0.89)	−0.20 (0.88)	−0.48 (0.89)	−0.04 (0.94)
*t*-test	−5.28	−5.30	2.10	5.95	0.37
*p*-value	<0.001	<0.001	0.04	<0.001	0.71

*Bold indicates component loadings >0.40*.

### Exploratory convergent validity associations

Exploratory correlation findings with the 5 PCA components are reported in Table 5, presented in Figure 4, and are described below.

**Table 5:** Convergent validity, disease course and specificity of PCA connectivity components.

	**rMDD > HC**		**rMDD < HC**		
**Characteristic^a^**	**1: Bilateral PCC with CCN**	**2: Right DMN with CCN/SEN**	**3: R-HPF with DMN**	**4: HPF with SMN**	**5: Bilateral PCC with DMN**
**CONVERGENT VALIDITY**					
RRS Total	0.19 [−0.05, 0.41]	0.38^**^ [0.15, 0.57]	−0.08 [−0.31, 0.16]	−0.29^*^ [−0.49, −0.05]	0.03 [−0.27, 0.27]
CC Factor	−0.21^†^ [−0.42, 0.03]	−0.12 [−0.34, 0.12]	−0.13 [−0.35, 0.11]	−0.13 [−0.35, 0.11]	−0.08 [−0.31, 0.16]
SLLT d'	−0.05 [−0.27, 0.17]	−0.01 [−0.23, 0.21]	0.23^*^ [0.01, 0.43]	−0.05 [−0.27, 0.18]	0.12 [−0.11, 0.33]
FEPT Happy Accuracy^b^	0.01 [−0.23, 0.24]	−0.04 [−0.27, 0.19]	−0.27^*^ [−0.47, −0.03]	−0.24^*^ [−0.45, −0.01]	−0.06 [−0.29, 0.18]
Sad Accuracy	−0.03 [−0.26, 0.20]	−0.11 [−0.33, 0.13]	−0.02 [−0.25, 0.22]	−0.10 [−0.32, 0.14]	−0.17 [−0.38, 0.07]
**DISEASE COURSE**					
HAM–D	0.24^*^ [0.02, 0.43]	0.10 [−0.12, 0.31]	0.06 [−0.16, 0.28]	−0.30^**^ [−0.48, −0.09]	0.04 [−0.18, 0.26]
Number of Episodes^b^	−0.07 [−0.22, 0.35]	−0.20 [−0.46, 0.09]	−0.15 [−0.42, 0.15]	0.09 [−0.21, 0.36]	0.02 [−0.27, 0.30]
Age at Onset^b^	−0.09 [−0.37, 0.20]	−0.07 [−0.35, 0.22]	0.17 [−0.12, 0.44]	0.08 [−0.22, 0.36]	0.10 [−0.19, 0.38]
**SPECIFICITY**					
Age	0.07 [−0.15, 0.28]	0.11 [−0.11, 0.32]	0.18 [−0.04, 0.38]	0.07 [−0.15, 0.28]	0.09 [−0.13, 0.30]
Verbal IQ	−0.02 [−0.24, 0.20]	0.01 [−0.21, 0.22]	–0.26^*^ [−0.45, −0.04]	–*0.19*^†^ [−0.39, 0.03]	−0.13 [−0.34, 0.09]

*PCA, Principle Components Analysis. Analyses included all subjects without missing data. Italics indicate a significant partial correlation after controlling for diagnosis (p < 0.05, one-tailed), which was only conducted following a significant initial correlation*.

a *Some cognitive features and clinical variables were significantly correlated: verbal IQ was negatively related to SLLT d' (r = −0.25, p = 0.02), and residual HAM–D was positively related to both RRS total (r = 0.25, p = 0.04) and FEPT sad accuracy (r = 0.29, p = 0.01). No cognitive features were significantly correlated with each other*.

b *Spearman's correlation ρ reported due to non-normal distribution*.

† *p < 0.10*,

* *p < 0.05*,

** *p < 0.01, all two-tailed*.

#### Rumination

Increased rumination was significantly associated with greater connectivity in Component 2, Right DMN with CCN/SEN (*r* = 0.38, *p* = 0.001, 95% CI [0.15, 0.57]), and less connectivity in Component 4, hippocampi with SMN (*r* = −0.29, *p* = 0.02, 95% CI [−0.49, −0.05]).

#### Cognitive control

Increased cognitive control showed a trend-level association with decreased connectivity of Component 1, Bilateral PCC with CCN (*r* = −0.21, *p* = 0.08, 95% CI [−0.42, 0.03]). No correlations reached significance for cognitive control and the other 4 connectivity components.

#### Facial emotion detection

Increasing accuracy for happy faces was associated with decreasing connectivity in Component 3, Right HPF with DMN (*r*_*s*_ = −0.27, *p* = 0.03, 95% CI [−0.47, −0.03]), and with decreasing connectivity in Component 4, hippocampi with SMN (*r*_*s*_ = −0.24, *p* = 0.05, 95% CI [−0.45, −0.01]). There were no significant or trend-level associations for accuracy for sad faces (all *p* > 0.10).

#### Memory

SLLT hit d' was significantly associated with Component 3, Right HPF with DMN (*r* = 0.23, *p* = 0.04, 95% CI [0.01, 0.43]), also significant after covarying for diagnosis (*r* = 0.24, *p* = 0.02, 95% CI [0.02, 0.44]).

### Exploratory disease course associations

Increased residual depression scores were associated with decreased connectivity in Component 4, hippocampi with SMN (*r* = −0.30, *p* = 0.01, 95% CI [−0.48, −0.09]), and increased connectivity in Component 1, Bilateral PCC with CCN (*r* = 0.24, *p* = 0.03, 95% CI [0.02, 0.43]). Number of episodes and age at onset of depression were not associated with any connectivity components (all *p* > 0.30).

### Exploratory specificity associations

Although both age and verbal IQ were carefully controlled across groups in this narrow age range, due to known continued brain development across young adulthood (49), both variables were examined in relation to connectivity components. Age was not significantly associated with any connectivity components (all *p* > 0.10). Verbal IQ was significantly negatively associated with Component 3, right hippocampus with DMN (*r* = −0.26, *p* = 0.02, 95% CI [−0.45, −0.04]), also significant after covarying for diagnosis (*r* = −0.26, *p* = 0.01, 95% CI [−0.45, −0.04]). Verbal IQ also showed a trend-level negative association with Component 4, hippocampi with SMN (*r* = −0.19, *p* = 0.09, 95% CI [−0.34, 0.03]), significant after covarying for diagnosis (*r* = −0.21, *p* = 0.03, 95% CI [−0.42, −0.01]).

### Exploratory state-independence comparisons

There were no significant connectivity differences between rMDD and HC groups in any of the ROIs identified as significant in active depression (18) or familial high-risk for depression (19) (all *p* > 0.10; see Figure 3B). Significant connectivity clusters between HC and rMDD in the current study within regions near dorsolateral prefrontal cortex are also displayed for comparison purposes (Figure 3 and Table 6).

**Table 6:** Current sample connectivity in regions implicated in active and familial risk for depression.

	**Estimated marginal effects**				**Diagnosis effect**		
	**HC**		**rMDD**				
**Connectivity**	***M***	***SD***	***M***	***SD***	**Direction**	***F***	***p***
**ACTIVE DEPRESSION SUPERIOR FRONTAL GYRUS^18^**							
Left Dorsal Nexus from L–PCC	0.17	0.02	0.20	0.02	rMDD > HC	0.88	0.35
Right Dorsal Nexus from L-PCC^b^	0.15	0.02	0.16	0.02	rMDD > HC	0.05	0.82
Left Dorsal Nexus from R-PCC^c^	0.16	0.02	0.18	0.02	rMDD > HC	1.03	0.31
Right Dorsal Nexus from R-PCC^b^	0.21	0.02	0.22	0.02	rMDD > HC	0.01	0.91
**FAMILIAL RISK FOR DEPRESSION MIDDLE FRONTAL GYRUS^19^**							
Left dlPFC from L-PCC	0.07	0.02	0.07	0.01	rMDD > HC	0.07	0.79
Right dlPFC from L-PCC	0.003	0.01	0.002	0.01	HC > rMDD	0.002	0.96
Left dlPFC from R-PCC	0.03	0.02	0.04	0.01	rMDD > HC	0.36	0.55
Right dlPFC from R-PCC	0.05	0.01	0.05	0.01	rMDD > HC	0.001	0.97
**REMITTED DEPRESSION DORSOLATERAL PREFRONTAL CORTEX CLUSTERS^a^**							
Right SFG from L-PCC	0.03	0.03	0.17	0.02	rMDD > HC	19.50	<0.001
Left MFG from L-PCC	−0.08	0.02	0.04	0.02	rMDD > HC	14.90	<0.001
Right MFG from L-PCC^d^	0.12	0.03	0.26	0.02	rMDD > HC	17.14	<0.001
Right SFG from R-PCC	0.15	0.03	0.28	0.03	rMDD > HC	12.64	0.001
Left MFG from R-PCC^c^	−0.03	0.02	0.08	0.02	rMDD > HC	13.51	<0.001
Right MFG from R-PCC	0.05	0.02	0.18	0.02	rMDD > HC	24.41	<0.001

*Estimated means, standard errors and significance determined using full linear mixed model with diagnosis, time, diagnosis by time, sex, and site as covariates*.

a *Current study's diagnostic clusters near dorsolateral prefrontal cortex*.

b *p < 0.05 for Site*.

c *p < 0.05 for Sex*.

d *p < 0.05 for Scan Session*.

Within linear mixed models for active depression ROIs, right superior frontal gyrus connectivity showed a significant fixed effect of site with bilateral PCC seeds [left: *F*_(1, 82)_ = 4.16, *p* < 0.05; right: *F*_(1, 82)_ = 6.50, *p* = 0.01], such that participants at UM had lower connectivity [left: B = −0.05 (0.03); right: B = −0.07 (0.03)]. In addition, left superior frontal gyrus connectivity showed a significant fixed effect of sex with right PCC seed [*F*_(1, 82)_ = 4.74, *p* = 0.03], such that males showed greater connectivity [B = 0.07 (0.03)]. No other fixed or random effects were significant (all *p* > 0.05). Within familial risk ROIs, there were no significant fixed or random effects (all *p* > 0.05).

## Discussion

DMN seed-to-cluster connectivity differences in rMDD young adults compared to healthy comparison subjects demonstrated modest or better reliability in 16 of 17 clusters, independent of sex, site, and movement deviation. From PCC seeds of the DMN, 10 clusters showed hyperconnectivity in rMDD compared to HC with bilateral posterior cingulate cortex and 7 primarily bilateral anterior hippocampal formation seeds showed hypoconnectivity in rMDD relative to HC.

The present results contribute to a series of studies with rs-fMRI suggesting that DMN biomarkers may be detectable with this technology, further demonstrating reliability in this technique. Previous studies suggest that DMN or DMN to dorsolateral prefrontal cortex connectivity are elevated in a symptom-independent manner (17, 18), and that this aberrant connectivity is related to familial risk (19). We also found elevated bilateral PCC seed with dorsolateral prefrontal cortex connectivity in remitted individuals. In fact, right DMN with dorsolateral prefrontal cortex connectivity was moderately reliable, significantly related to rumination but not depressive symptoms, and was invariant to time, sex, movement translations, site parameters, and scanner sequences. Thus, not only does this exploratory finding corroborate clinical meaningfulness and course-invariance, it may also meet the reliability criterion put forth by Gottesman and Gould (1) and Gould and Gottesman (2) for a candidate endophenotype.

Most of the time-invariant clusters found showed moderate to adequate reliability, a notable advance in demonstrating reliability in functional neuroimaging techniques. DMN reliability is consistent with work in healthy samples demonstrating reliability of functional connectivity in several task and resting-state scans (50). Here, reliability over a period of one to several months (i.e., test-retest reliability) is a rigorous test of time-invariance or temporal stability. The cluster with lowest reliability in all individuals, showing hypoconnectivity between right HPF and paracentral lobule, loaded onto Component 4, HPF with SMN, which was negatively associated with residual depression symptoms. Given alignment with previous research (51), these symptom-related connectivity patterns, even at the remitted phase, might be important as targets for treatment-resistant MDD in secondary prevention trials (15).

The most predominant pattern of diagnostic differences was inter-hemispheric hyperconnectivity of the core, dorsal DMN subsystem with CCN and hypoconnectivity across ventrolateral subsystems of DMN, with SEN, and with the SMN (12). Disease may divert development of optimal within-network and inter-hemispheric neurodevelopment and homogeneity. CCN prefrontal cortex connectivity is particularly implicated in the pathophysiology of MDD, thought to dysfunctionally regulate several networks in depression [e.g., (18)]. We found 6 interhemispheric prefrontal clusters that exhibited increased connectivity in rMDD across DMN and CCN networks. Although slightly different regions and networks are identified than in currently depressed and familial at-risk groups, these clusters highlight regions identified as symptom-invariant in an active depression cohort (17). Due to potential state-invariance (17, 18), these particular connectivity patterns are not likely to be compensatory mechanisms. Instead, they may reflect relatively small fluctuations in trait rumination or impulsivity between phases of illness.

Exploratory correlation analyses after data reduction highlight convergence with cognitive features of depression risk/history. PCA data reduction showed 2 components linking DMN and CCN nodes (hyperconnectivity), 2 components showing connectivity across subsystems of the DMN (hypoconnectivity), and 1 component connecting the medial temporal DMN subsystem with SMN nodes. Three of these components showed significant convergent validity with other cognitive features of illness.

What initially appear to be paradoxical findings of DMN hyper- versus hypo-connectivity in the literature in remitted versus active depression may be the expression of separate characteristics of the disorder and nuanced representations of DMN subcomponents with distinct disease-to-biomarker relationships [e.g., (52, 53)]. Indeed, several studies have begun to dissociate disease-related effects of these subcomponents of DMN in the pathophysiology of depression (11, 54, 55). Further complicating these comparisons is the knowledge that stage of illness (age, number of episodes) and presence of active symptoms [active vs. remitted, e.g., (17)] do change connectivity results. Results from the present study suggest those phenotypes related to the experience of sustained, negative emotion and regulation thereof, were reflected by hyperconnectivity with posterior cingulate, in line with numerous other studies in active depression [e.g., (10, 13)]. In contrast, cognitive features typically associated with medial temporal function, such as emotion processing and memory, were related to hypoconnectivity in remitted MDD, consistent with findings in subthreshold depression (40).

Our results encourage work on whether there may be 2, or even 3 potential DMN “pathologies” of depression, acting on differing aspects of brain and behavior that better explain disparate findings in the field. For example, hypoconnectivity with HPF associated with decreased memory performance and increased emotion identification may result from one pathology, while hyperconnectivity with PCC associated with decreased cognitive control and increased rumination may result from another. Several other groups have also reported distinct separate associations and effects in depression for each DMN subnetwork, although these have found in active depression a dorsal DMN hypoconnectivity associated with autobiographical memory and a ventral DMN hyperconnectivity associated with rumination (11, 54, 55). Discordance with the current findings may reflect methodological differences (independent component analysis compared to our seed-to-node analysis; autobiographical memory compared to semantic list learning), or masking/distorting of trait-like biomarkers by the active state of depression. Indeed, Li et al. (16) reported a divergence in anterior and posterior DMN subnetworks after treatment response, suggesting that symptom reduction from active depression to remission may affect each DMN subnetwork differently for individuals with this disorder.

An alternative hypothesis that may explain these apparent discrepancies is that one DMN pattern may be reflective of disease risk (hyperconnectivity) and the other of disease scar (hypoconnectivity). Consistent with this alternative interpretation, cognitive control and rumination weaknesses tend to precede depression in high risk samples, whereas emotion perception and memory deficit results are more mixed, (56, 57) and may develop as the disease progresses. Moreover, differences in connectivity that are observed in remission may reflect compensation to maintain wellness (58).

Limitations and strengths are present in this work. There were significant associations with residual depressive symptoms in the PCC component related to cognitive control and the HPF component related to rumination, suggesting concomitant fluctuations between depressive symptoms, cognitive control, rumination and aberrant connectivity of these DMN components. Similarly, verbal intelligence was significantly associated with hippocampal DMN and SMN hypoconnectivity. While these associations weaken the performance-based specificity of aberrant connectivity in rMDD, they also reflect potential residual disease processes that may increase risk for recurrence and highlight the need for fine-tuned measurement of fluctuations in connectivity, depressive symptoms, and performance markers over time. In addition, it is possible that symptoms, including sleep disruptions, reflect or distort some of the observed effects (59). The current study's modest sample size and dissimilitude with another subcomponent DMN analysis in active MDD (54) also reflect the need to recruit and follow larger samples over time to fully address these important questions about reliability and state-independence. Indeed, these results underline the sensitivity of functional connectivity-related analyses to context and phase of illness. Finally, the modest nature of these exploratory relationships with cognitive features, while offering some clinical meaning, only partially explain connectivity differences and do not survive corrections for multiple comparisons.

The current findings were robust against known site differences such as scanner type and sequencing, verbal IQ, and race, despite potential multifactorial site effects that were only controlled for as a covariate in the models reported herein (29). Importantly, site and scanner differences tend to introduce more variability in analytic results, thus the current findings could be perceived as a more conservative test of reliability or endophenotypic candidate relationships. The current study is unable to tease apart risk from disease scar/effect; disrupted connectivity may reflect a stable scar from previous depressive episodes. Absent any longitudinal study with testing in both the acute and remitted stages of depression, the current study can only compare against other studies regarding the state independence of the current findings. Notably, the current study recruited a young adult sample within a very narrow age window to limit the variability imposed by development and disease course, thus it is unknown whether the current findings reflect aberrant connectivity in rMDD throughout the lifespan, in the context of antidepressant treatment or if these patterns change across development and aging.

In conclusion, the present study adds time-invariance and reliability as important characteristics of DMN functional connectivity, most notably PCC to dorsolateral prefrontal cortex, as a potential endophenotype for depression. Within an rMDD sample, generally adequate individual level reliability was demonstrated for 11 hyper- and 6 hypo-connectivity seed-node connections, within and across-networks, and within and across-hemispheres. Many of these connectivity patterns were associated with known cognitive markers of depression as well as current residual symptoms. Hyperconnectivity between DMN and prefrontal cortex CCN regions and hypoconnectivity within medial temporal DMN may represent stable, trait-based endophenotypes of remitted depression course.

## Ethics statement

This study was carried out in accordance with the recommendations of the NIMH and University of Michigan (UM) and University of Illinois at Chicago (UIC) Institutional Review Boards with written informed consent from all subjects. All subjects gave written informed consent in accordance with the Declaration of Helsinki. The protocol was approved by both UM and UIC Institutional Review Boards.

## Author contributions

Study design and planning by SL, MM, and JKZ. Data collection carried out by KB, LJ, KS, SD, and SL; data modeling and analysis carried out by KB, LJ, KS, JG, SD, RJ, OA, SL; and writing/editing carried out by all authors. SL and KB had full access to all the data in the study and take responsibility for the integrity of the data and the accuracy of the data analysis.

### Conflict of interest statement

The authors declare that the research was conducted in the absence of any commercial or financial relationships that could be construed as a potential conflict of interest.
